# Supplementary figures and images for: Chemokine Regulation in Temporomandibular Joint Disease: A Comprehensive Review
Source: Genes (Basel). 2023 Feb 4;14(2):408. doi: 10.3390/genes14020408 (PMC9956915; doi:10.3390/genes14020408)

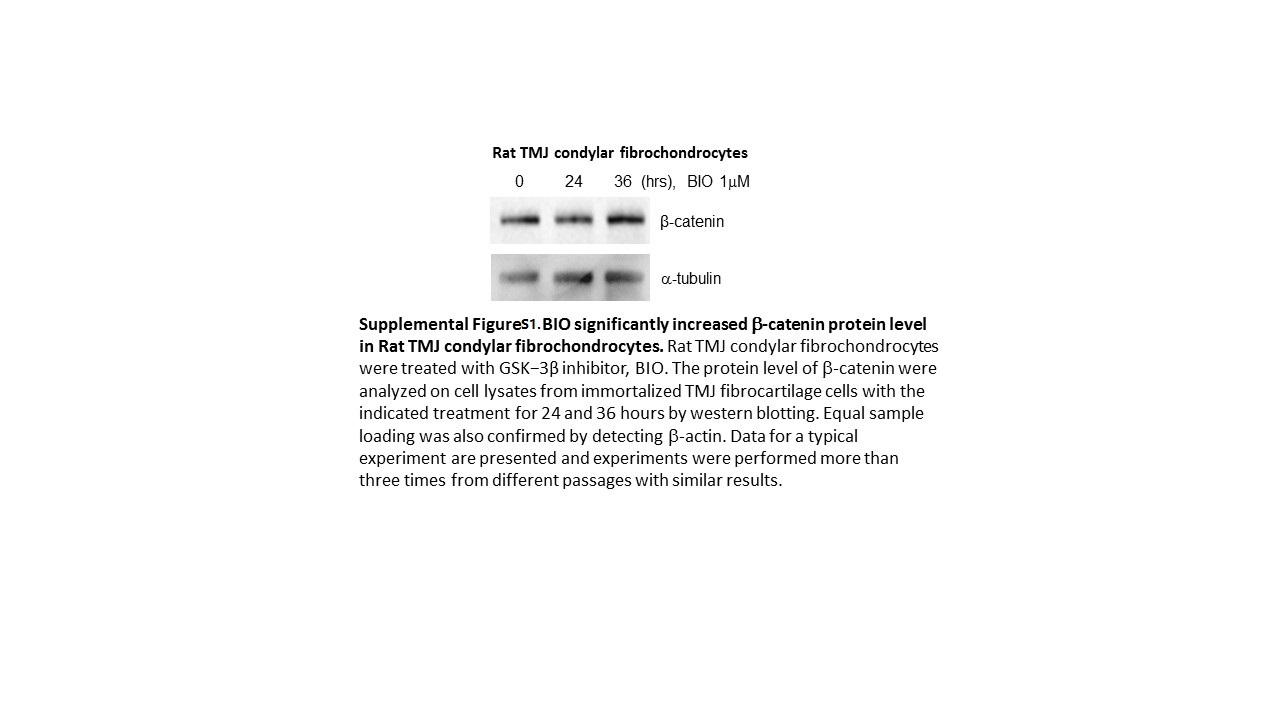

Supplement: Supplementary file 1 [file genes-14-00408-s001.zip › genes-2081041-supplementary.JPG]
